# Supplementary material for: Predicting visual function by interpreting a neuronal wiring diagram
Source: Nature. 2024 Oct 2;634(8032):113–23. doi: 10.1038/s41586-024-07953-5 (PMC11446822; doi:10.1038/s41586-024-07953-5)
Supplement: Supplementary file 6 — CRF and ERF predictions for individual TmY4 and TmY9 cells. Analogous to Supplementary Data 3, but for TmY target types. Shown are the top four monosynaptic pathways, the strongest pathway passing through each of the top ten intermediary types (ranking from Extended Data Fig. 7), and the trisynaptic pathway Tm1–TmY–Dm3–TmY (see the section entitled Prediction of spatial normalization). [file 41586_2024_7953_MOESM6_ESM.zip › DataS4/TmY9qΓèÑ/720575940612661873.pdf]

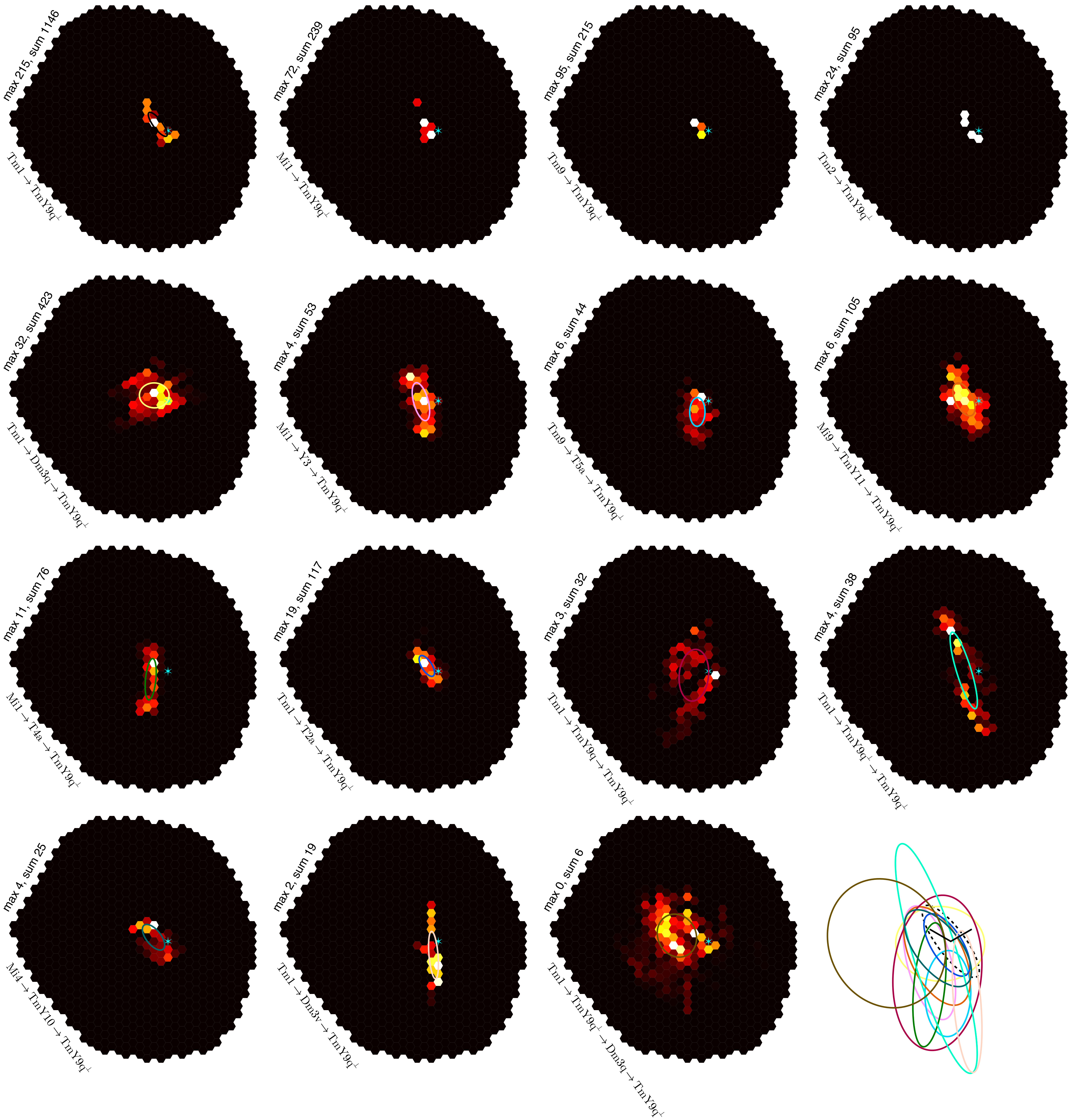
max 215, sum 1146
Tm1 → TmY9q⊥
max 72, sum 239
Mi1 → TmY9q⊥
max 95, sum 215
Tm9 → TmY9q⊥
max 24, sum 95
Tm2 → TmY9q⊥
max 32, sum 423
Tm1 → Dm3q → TmY9q⊥
max 4, sum 53
Mi1 → Y3 → TmY9q⊥
max 6, sum 44
Tm9 → T5a → TmY9q⊥
max 6, sum 105
Mi9 → TmY11 → TmY9q⊥
max 11, sum 76
Mi1 → T4a → TmY9q⊥
max 19, sum 117
Tm1 → T2a → TmY9q⊥
max 3, sum 32
Tm1 → TmY9q⊥ → TmY9q⊥
max 4, sum 38
Tm1 → TmY9q⊥ → TmY9q⊥
max 4, sum 25
Mi4 → TmY10 → TmY9q⊥
max 2, sum 19
Tm1 → Dm3v → TmY9q⊥
max 0, sum 6
Tm1 → TmY9q⊥ → Dm3q → TmY9q⊥
